# *Leptospira* Serovar as Prognostic Factor

**DOI:** 10.3201/eid1608.100520

**Published:** 2010-08

**Authors:** Motoi Suzuki, Koya Ariyoshi

**Affiliations:** Nagasaki University, Nagasaki, Japan

**Keywords:** *Leptospira interrogans* serovar Icterohemorrhagiae, leptospirosis, bacteria, letter

**To the Editor**: Herrmann-Storck et al. (*1*) investigated prognostic factors of leptospirosis and concluded that infection with *Leptospira interrogans* serovar Icterohemorrhagiae was linked to severe outcomes. We have concerns about this conclusion.

These researchers were comparing clinical severity of disease among patients for whom serovars of infecting isolates had been identified. However, in that study, blood culture was performed for only 88 (52%) of 168 case-patients, and serovars were identified for 40 (73%) of 55 *Leptospira* strains isolated. Expecting these 40 patients (24% of total) to represent all case-patients in the study is unjustified.

Also, the authors evaluated potential risk factors among these patients by applying a multivariable logistic regression model. This process is questionable. First, the sample size of 40 is not large enough to warrant multivariable analysis with 9 independent variables. Actually, only 8 case-patients had severe disease, although at least 10 outcomes are required for variables in a logistic regression model (*2*).

Second, the model selected is inappropriate. Variables such as thrombocytopenia, hyperneutrophilia, hyperamylasemia, and elevated asparate aminotransferase levels are laboratory findings of severe leptospirosis (and not risk factors of disease). These factors should not be included in a multiple logistic regression model as confounders. We believe it is premature to reach a conclusion about the association between *Leptospira* serovars and clinical severity from the data presented by Herrmann-Storck et al.
