# Supplementary material for: Leptospira Serovar as Prognostic Factor
Source: Emerg Infect Dis. 2010 Aug;16(8):1333. doi: 10.3201/eid1608.100520 (PMC3298314; doi:10.3201/eid1608.100520)
Supplement: Related texts

# Reply

**DOI:** 10.3201/eid1608.100763

**Authors:** Cécile Herrmann-Storck

**Affiliations:** Central University Hospital, Pointe-à-Pitre, Gaudeloupe, France

**In response:** We thank Suzuki and Ariyoshi for their letter (*1*). Identification of serovar and species can only be accomplished by isolating *Leptospira* strains, and obtaining isolates and identifying the serovar and species are especially difficult for human cases of leptospirosis. The bacteria are present in blood for only 1 week after the onset of the disease; they also are fastidious and difficult to grow. Serologic testing gives only a possible serogroup, but it is the only diagnostic tool for confirming patients’ infection after 1 week of disease. A strength of our study (*2*) is that it contains extensive epidemiologic, clinical, and biological data and provides a broad collection of identified strains.

Including laboratory findings such as thrombocytopenia, hyperneutrophilia, and hyperamylasemia in the model was appropriate for the following reasons. First, they were not used to establish the definition of severity. Second, the variables included in the model were defined, not according to the norms but at a given level far above the norms (thrombocytopenia <50 g/L, hyperneutrophilia >12 g/L, amylase >285 U/L), which have been recently suggested as possible predictors of severity in other reports.

We are aware that the statistical model has its limits in the context of this retrospective study. We must point out that the conclusion of the independent involvement of *Leptospira* serovar Icterhoaemorragiae in severity is made in the context of Guadeloupe with its particular ecology and insular features, and results were compared with those for given cocirculating strains that are sometimes different in other areas of the world. The real implication of our study is the opportunity it presents to explore some virulence factors in this particular serovar, to compare the results with those of other studies conducted in other areas with the same tools of identification, and to pave the way for a much larger prospective study in the region.

## References

[1] Suzuki M, Ariyoshi K. *Leptospira* serovar as prognostic factor. Emerg Infect Dis. 2010;16:1333. 10.3201/eid1608.10052020678345PMC3298314

[2] Herrmann-Storck C, Saint-Louis M, Foucand T, Lamaury I, Deloumeaux J, Baranton G, Severe leptospirosis in hospitalized patients, Guadeloupe. Emerg Infect Dis. 2010;16:331–4.2011357410.3201/eid1602.090139PMC2957992
